# Development and Validation of a Quantitative LC-MS/MS Method for Measuring CYP4V2 Enzyme Activity via 12-Hydroxylauric Acid in rAAV-hCYP4V2 Gene Therapy Products

**DOI:** 10.3390/molecules31091417

**Published:** 2026-04-24

**Authors:** Ge Ren, Xi Qin, Yiran Li, Wenhong Fan, Wenjing Luo, Yanrong Cao, Yang Wang, Yong Zhou, Chenggang Liang

**Affiliations:** 1 National Institutes for Food and Drug Control, Beijing 100050, China; renge@hbut.edu.cn (G.R.); qinxi@nifdc.org.cn (X.Q.); yiranli202402@163.com (Y.L.); fanwh@nifdc.org.cn (W.F.); 2 State Key Laboratory of Drug Regulatory Science, Beijing 100050, China; 3 School of Life and Health Sciences, Hubei University of Technology, Wuhan 430068, China; 4 Shanghai Vitalgen BioPharma Co., Ltd., Shanghai 201210, China; wj.luo@vitalgen.com (W.L.); yr.cao@vitalgen.com (Y.C.)

**Keywords:** LC-MS/MS, CYP4V2, enzyme activity, AAV, gene therapy, lauric acid ω-hydroxylation, method validation

## Abstract

Bietti crystalline dystrophy (BCD) is a hereditary retinal disease caused by loss-of-function mutations in the *CYP4V2* gene. Gene replacement therapy using rAAV-hCYP4V2 represents a promising therapeutic strategy, requiring robust bioassays for product quality control. This study developed and validated a sensitive LC-MS/MS method for quantifying CYP4V2 enzyme activity. Lysates from HeLa-AAVR cells transduced with rAAV-hCYP4V2 (MOI = 3 × 10^5^) were used, with lauric acid as substrate supplemented with cytochrome P450 reductase, cytochrome b5, and NADPH. The ω-hydroxylated product (12-hydroxy lauric acid) was quantified using tolbutamide as an internal standard. Method validation followed ICH guidelines. Results demonstrated excellent specificity with negligible background in negative controls. Linearity was achieved over 0.5–100 ng/mL (*R*^2^ > 0.99), with an average recovery of 100.6%. Intra-batch and inter-batch precision RSDs were <47.8% and <28.4%, respectively. Product stability was maintained for ≥4 weeks at −80°C. The method was successfully applied to three AAV serotypes (AAV2, AAV8, and AAV2/8), with all RSDs < 23.9%. This validated LC-MS/MS bioassay provides a crucial quality control tool for potency assessment, process development, batch release, and stability studies of rAAV-hCYP4V2 gene therapy products.

## 1. Introduction

Bietti crystalline dystrophy (BCD) is an autosomal recessive hereditary retinal degenerative disease [1,2,3,4]. Its pathological characteristics include the presence of shiny yellow crystalline deposits in the retinal pigment epithelium (RPE) and choroid [5], accompanied by progressive atrophy of RPE and photoreceptor cells, which can ultimately lead to visual field defects, night blindness, and loss of central vision [6]. Molecular genetic studies have shown that BCD is primarily caused by loss-of-function mutations in the *CYP4V2* gene. This gene encodes the CYP4V2 enzyme, a member of the cytochrome P450 superfamily, whose primary physiological function is to catalyze the ω-hydroxylation of medium- and long-chain fatty acids, including palmitic acid and lauric acid [7]. The CYP4V2 protein is mainly expressed in retinal pigment epithelial (RPE) cells and demonstrates ω-hydroxylase activity toward ω-3 polyunsaturated fatty acids such as docosahexaenoic acid (DHA) and eicosapentaenoic acid (EPA) [5,8]. Targeted lipidomic studies have revealed that BCD patients exhibit significantly decreased plasma levels of ω-3 and ω-6 long-chain polyunsaturated fatty acids (DHA, EPA, and arachidonic acid) and their downstream eicosanoid metabolites via cyclooxygenase and lipoxygenase pathways [9]. Inactivation of the CYP4V2 enzyme can obstruct fatty acid metabolism, triggering abnormal lipid accumulation, oxidative stress, and ferroptosis [10], ultimately resulting in irreversible damage to RPE cells. Currently, there is no approved, effective therapy for BCD, resulting in a significant unmet clinical need [11].

Gene replacement therapy using adeno-associated virus (AAV) vectors offers a promising therapeutic approach for BCD [12]. rAAV-hCYP4V2 is a gene therapy product under development based on AAV vectors, designed to deliver the functional *CYP4V2* gene to retinal cells via a single administration, thereby restoring enzyme function and delaying or preventing disease progression. With a coding sequence of 1578 bp, *CYP4V2* can be effectively encapsulated into AAV vectors, and RPE cells are suitable targets for AAV-mediated gene augmentation therapy [5]. Currently, three products (NCT06302608, NCT04722107, and NCT05694598) are undergoing clinical trials, all of which have shown significant progress [2,11,13]. Notably, a phase 1/2 clinical trial of rAAV2-hCYP4V2 (NGGT001) published in 2025 reported no serious safety concerns and a mean improvement of 13.9 letters in best-corrected visual acuity in treated eyes at 12 months post-treatment [2]. Two products have entered Phase III clinical trials (ZVS101e and VGR-R01), representing significant advances in BCD gene therapy development [5]. To ensure the batch-to-batch consistency, potency, and effectiveness of such biological products during production and quality control, it is crucial to establish a precise, stable, and quantifiable method for assessing biological activity [14]. Potency is a critical quality attribute (CQA) for AAV-based gene therapy products, and regulatory guidelines from the FDA and EMA require that potency assays reflect the product‘s mechanism of action (MoA) and be validated for product release, stability testing, and comparability studies [15].

At present, commonly used CYP450 enzyme activity assays, such as in vitro tissue homogenization and fluorescence-based methods, suffer from limitations including indirect inference, susceptibility to matrix interference, or insufficient specificity for distinguishing target enzyme activity. Thus, they fail to meet the requirements for efficient potency determination of gene therapy products [16,17]. Due to its high sensitivity, specificity, and excellent quantitative performance, liquid chromatography-mass spectrometry (LC-MS/MS) has become the gold standard for quantifying low-concentration small-molecule metabolites in complex biological matrices. However, no systematically optimized and comprehensively validated LC-MS/MS method is currently available for quantifying CYP4V2 enzyme activity in rAAV-hCYP4V2 gene therapy products that can be directly used for product quality control and release.

Therefore, this study aims to establish and validate an LC-MS/MS-based CYP4V2 enzyme activity assay to directly, specifically, and quantitatively evaluate the functional activity of rAAV-hCYP4V2. This method utilizes lauric acid as the substrate, and the enzymatic catalytic activity is reflected by the generated ω-hydroxylation product (12-hydroxy lauric acid). Systematic optimization and comprehensive validation of the method were performed to ensure compliance with the technical requirements for biological product activity determination specified in the Pharmacopoeia of the People’s Republic of China and relevant international guidelines. The developed method represents a reliable analytical tool for the research, development, and quality control of rAAV-hCYP4V2 products.

## 2. Results

### 2.1. Exploration of the Enzymatic Reaction System: Establishment of the System from Intracellular to Extracellular Reaction

In the early stages of the study, we added substrates directly to FreeStyle 293 or ARPE-19 cells transfected with BCD plasmids to initiate the reaction; however, no reaction products were detected (concentrations below the lower limit of quantification (LLOQ)). This indicates that substrate accessibility or endogenous metabolic interference may limit reaction efficiency in intact cells. Therefore, the research strategy was shifted to an extracellular reaction system, in which cell lysate was used as the enzyme source to reconstruct an in vitro reaction environment containing all necessary cofactors.

Preliminary experiments revealed that when lysates from 293 cells transfected with the BCD plasmid were used, and excess cytochrome P450 reductase (40 nM) and cytochrome b5 (20 nM) were added, the product 12-hydroxylauric acid could be successfully detected using 250 μM lauric acid as the substrate. The yield of this product showed a good linear relationship with reaction time over 90 min (*R*^2^ = 0.970). This result confirms the feasibility of the extracellular reaction strategy and highlights the crucial role of cofactor saturation in enabling the CYP4V2 enzyme to reach its maximum catalytic rate. Based on this finding, the core reaction conditions for subsequent method development were defined as follows: cell lysate as the enzyme source and an optimized reaction system containing 40 nM P450 reductase, 20 nM cytochrome b5, and 250 μM lauric acid.

### 2.2. Optimization of Cell Models and Virus Transduction Conditions

#### 2.2.1. Exploration of MOI in Viral Transduction

**Figure 1:**
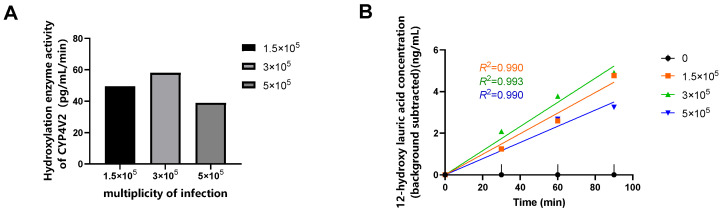
(**A**) CYP4V2 hydroxylase activity in HEK293 cells transduced with rAAV-hCYP4V2 at different MOIs over 0 to 90 min. (**B**) Changes in product concentration over time (0 to 90 min) following rAAV-hCYP4V2 transduction in HEK293 cells at different MOIs. Concentrations are presented after background subtraction (subtraction of the 0-min time point value) to correct for endogenous metabolites. The fitted lines were constrained to pass through the origin. Data are from a single representative optimization experiment.

Virus transduction was performed in HEK293 cells seeded at a density of 3 × 10^6^ cells, and enzyme activity was assessed at reaction times of 0, 30, 60, and 90 min. When the MOI was 3 × 10^5^, the linear relationship between enzymatic reaction and time was most pronounced, and the highest relative enzyme activity was observed (Figure 1). Therefore, this MOI value was selected for subsequent optimization.

#### 2.2.2. Optimization of Cell Types

**Figure 2:**
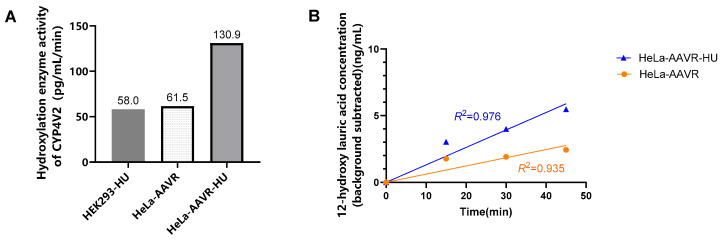
(**A**) CYP4V2 hydroxylase activity following rAAV-hCYP4V2 transduction in HEK293 and HeLa-AAVR cells. (**B**) Changes in product concentration over time following rAAV-hCYP4V2 transduction in HeLa-AAVR cells. Concentrations are presented after background subtraction (subtraction of the 0 min time point value) to correct for endogenous metabolites. The fitted lines were constrained to pass through the origin. Linear regression analysis confirmed a statistically significant slope for both groups (HeLa-AAVR: *p* = 0.007; HeLa-AAVR-HU: *p* = 0.002). Data are from a single representative optimization experiment.

To further improve viral transduction efficiency, HeLa-AAVR cells overexpressing the AAV receptor were used. Comparative experiments revealed that CYP4V2 enzyme activity was significantly higher in cells pretreated with 0.5 mM hydroxyurea (HU) than in cells without HU pretreatment or in HEK293 cells pretreated with 0.5 mM HU (Figure 2A). Therefore, the standard condition was defined as HeLa-AAVR cells pretreated with 0.5 mM HU for 24 h. Meanwhile, experimental results (Table S1) showed that the reaction products could be detected during the enzyme activity assay over a period of 0–120 min. Product concentration increased linearly with reaction time over the 0–45 min interval. Simple linear regression analysis with the intercept constrained to zero confirmed a statistically significant linear relationship for both the HeLa-AAVR group (*R*^2^ = 0.935, slope *p* = 0.007) and the HeLa-AAVR-HU group (*R*^2^ = 0.976, slope *p* = 0.002). The slope of the regression line—representing the initial reaction velocity—was approximately 2.1-fold higher in HU-pretreated cells (130.9 pg/mL/min) compared with untreated cells (61.5 pg/mL/min), consistent with the enhanced transduction efficiency observed in Figure 2A. Thus, in subsequent experiments, the enzymatic reaction was controlled within 45 min.

#### 2.2.3. Matrix Screening for Optimal Viral Transduction Conditions

**Figure 3:**
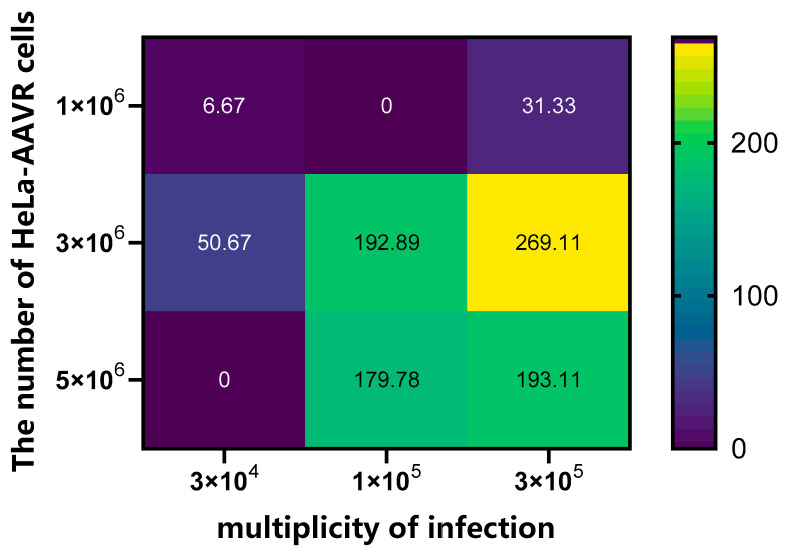
CYP4V2 hydroxylase activity detected by the endpoint method under different combinations of cell seeding number and MOI.

To further determine the optimal viral transduction conditions, product concentration was measured using a matrix experiment (cell seeding numbers: 1 × 10^6^, 3 × 10^6^, and 5 × 10^6^; MOI: 3 × 10^4^, 1 × 10^5^, and 3 × 10^5^) combined with an endpoint assay (reaction time: 45 min). The results showed that the highest enzyme activity (269.3 pg/mL/min) was achieved at a cell seeding number of 3 × 10^6^ and an MOI of 3 × 10^5^ (Figure 3, Table S2), which were therefore selected as the optimal transduction conditions. It should be noted that after background subtraction, some combinations resulted in calculated negative enzyme activity values. These negative values are a mathematical result of minor signal fluctuations around the background level or when the measured signal falls below the lower limit of quantification (LLOQ). They do not represent actual negative enzymatic activity but rather indicate that the product concentration was indistinguishable from the background noise under these suboptimal transduction conditions.

#### 2.2.4. Confirmation of Enzyme Activity Reaction Time Points

**Figure 4:**
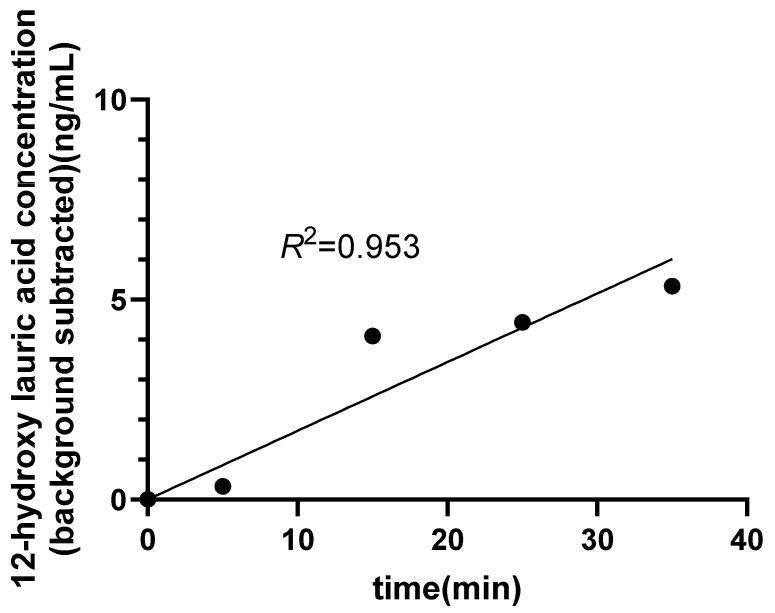
Concentrations of 12-hydroxylauric acid detected at different enzymatic reaction times (0–35 min). Concentrations are presented after background subtraction (subtraction of the 0 min time point value) to correct for endogenous metabolites. The fitted lines were constrained to pass through the origin. Data are from a single representative optimization experiment. Each dot represents a single measured concentration value.

The optimal time points for the enzymatic reaction were determined among 0, 5, 15, 25, 35, 45, 55, 65, 90, and 120 min. As shown in Figure 4, the product concentration reached its maximum at 35 min and exhibited a good linear relationship over the 0–35 min interval, after which the product concentration began to decrease (Table S3). Therefore, 0, 5, 15, 25, and 35 min were selected as the optimal time points for subsequent enzymatic reaction assays.

### 2.3. Optimization of the LC-MS/MS Detection Method

During the early stage of method development, methanol was used for standard curve preparation, and quantification was performed using the external standard method. This approach resulted in high background signals and substantial data fluctuation, indicative of a pronounced solvent effect. Subsequently, standard curve preparation was optimized using a blank matrix, specifically, blank cell lysate without viral transduction, and samples were pretreated simultaneously. Tolbutamide was then introduced as an internal standard, and quantification was performed using the internal standard method. Tolbutamide was selected as the internal standard because it contains a weakly acidic sulfonylurea group (pKa ≈ 5.3) that undergoes efficient deprotonation in negative ion ESI mode, similar to the carboxylic acid group of 12-hydroxylauric acid (pKa ≈ 4.8). This ensures comparable ionization behavior and consistent tracking of the analyte during liquid-liquid extraction and LC-MS/MS detection. The optimized method significantly improved assay reproducibility and accuracy.

**Figure 5:**
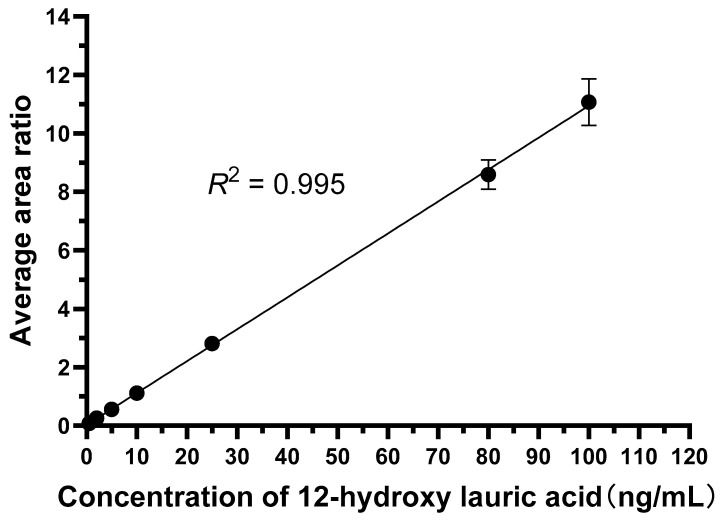
LC-MS/MS calibration curve. Data points represent the mean ± SD of three independent replicate analyses.

**Figure 6:**
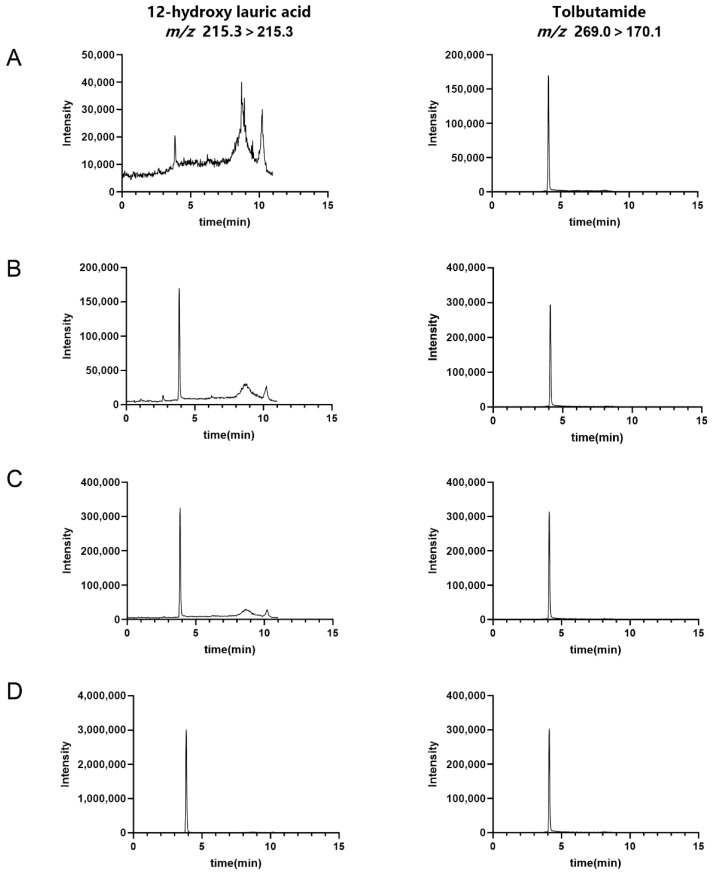
Representative LC-MS/MS chromatograms of 12-hydroxylauric acid and tolbutamide acquired in negative ion mode. The quantifier transition for 12-hydroxylauric acid was the pseudo-MRM *m*/*z* 215.3 → 215.3; tolbutamide (internal standard) was monitored via the characteristic fragment ion transition *m*/*z* 269.0 → 170.1. (**A**) 0.5 ng/mL (LLOQ), (**B**) 5 ng/mL, (**C**) 10 ng/mL, and (**D**) 100 ng/mL 12-hydroxylauric acid.

Under the optimized LC-MS/MS conditions, both 12-hydroxylauric acid and the internal standard produced sharp, symmetrical chromatographic peaks with good separation. The established calibration curve showed good linearity over the concentration range of 0.5–100 ng/mL (*R*^2^ > 0.99), with an LLOQ of 0.5 ng/mL, fully meeting the quantitative requirements for enzymatic reaction products. Figure 5 and Figure 6 show the calibration curve and representative spectra of 12-hydroxylauric acid and tolbutamide, respectively.

### 2.4. Method Validation and Application Outcomes

#### 2.4.1. Specificity

As shown in Table S4, the product concentrations in both the blank control group and the substrate (acetic acid) group were below the LLOQ. The highest product concentration was observed in the positive group (5.46 ng/mL after background subtraction), corresponding to an enzyme activity of 150.2 ± 9.430 pg/mL/min. The RSD within groups was 6.28%.

#### 2.4.2. Accuracy

According to the operation procedure, assuming the measured concentrations were x1 and x2, accuracy was calculated using the following formula: {$100\;+\;\frac{2x2\;-\;x1-\;\mathrm{QC}}{x1\;+\;\mathrm{QC}}$} × 100%. As shown in Table S5, spiked recovery accuracy ranged from 87.3% to 106.1%, with an average accuracy of 100.6% and a standard deviation of 6.045.

#### 2.4.3. Precision

Repeatability results are summarized in Table S6, with an RSD of 47.78% calculated from six replicates. Intermediate precision was determined by two operators who measured enzyme activity from three different virus batches over 2 days. The results are shown in Tables S6 and S7, with an RSD of 28.44% (dish 7–15).

#### 2.4.4. Linearity and Range

After linear regression was performed separately on each experimental group, the *R*^2^ value was obtained, with an average value ± standard deviation of 0.992 ± 0.007. As shown in Table S8, the LC-MS/MS method had good linearity. The calibration curve covered a concentration range of 0.5–100 ng/mL.

#### 2.4.5. Durability

The impact of different cell passages and rAAV-hCYP4V2 batches on enzyme activity measurement was evaluated in combination with precision testing. Data from the first to ninth cell culture dishes in the precision assessment were statistically analyzed, covering three HeLa-AAVR cell passages (P16, P20, and P19). The calculated enzyme activity was 130.2 ± 54.83 pg/mL/min, with an RSD of 42.11%. Data from the seventh to fifteenth cell culture dishes in the precision assessment were statistically analyzed, encompassing three rAAV-hCYP4V2 batches (V1, V2, and V3). The calculated enzyme activity was 141.3 ± 40.19 pg/mL/min, with an RSD of 28.44%.

After completion of the enzyme reaction, the product concentration was measured immediately and again after storage at −80 °C for 4 weeks. The ratio of enzyme activity measured after 4 weeks of storage to that measured immediately was calculated as the recovery rate, and its value was used to evaluate stability. As shown in Table S9, enzyme activity recovery rates of 101.8%, 111.3%, and 92.2% were obtained, indicating good stability of the method and the samples.

### 2.5. Practical Application of the Established Method

To evaluate the feasibility of the established method in practical applications, it was applied to determine the enzyme activity of three rAAV-hCYP4V2 products. As shown in Table 1, CYP4V2 enzyme activity was successfully detected in all three products, demonstrating that the method can be applied to different serotype vectors. Specifically, the average enzyme activity of the rAAV8-hCYP4V2 product was 328.4 ± 78.51 pg·mL^−1^·min^−1^ (RSD = 23.91%). The average enzyme activity of the rAAV2-hCYP4V2 product was 150.7 ± 24.26 pg·mL^−1^·min^−1^ (RSD = 16.10%), indicating excellent repeatability. The enzyme activity of the hybrid serotype product rAAV2/8-hCYP4V2 was 159.7 ± 34.39 pg·mL^−1^·min^−1^ (RSD = 21.53%).

## 3. Discussion

The LC-MS/MS method established in this study achieves, for the first time, direct and absolute quantification of CYP4V2 enzyme activity in rAAV-hCYP4V2 gene therapy products. Compared with traditional fluorescence- or immunology-based methods, this approach offers higher specificity and sensitivity and effectively minimizes matrix interference, making it suitable for detecting enzymes expressed at low levels. Through systematic optimization of the cell model (HeLa-AAVR + HU pretreatment), transduction conditions (MOI = 3 × 10^5^, cell number = 3 × 10^6^), and the enzymatic reaction system (coenzyme saturation conditions), the robustness and repeatability of the detection were significantly improved.

Verification results demonstrated that the method has good linearity over the range of 0.5–100 ng/mL, with high accuracy (average recovery rate of 100.6%), and that its precision meets the requirements of biological analytical methods. The intra-batch relative standard deviation (RSD ≤ 47.8%) falls within the commonly accepted range for biological activity assays, primarily due to inherent variability associated with cell-based experiments. Unlike chemical analyses, enzyme activity measured using this method is directly affected by multiple biological factors, such as virus transduction efficiency, cell status and passage number, lysis homogeneity, and fluctuations in the expression and function of the target enzyme. Therefore, the observed intra-batch variation reflects not only errors in the analysis process but also the inherent fluctuations of complex biological processes, from living cells to functional proteins. Nevertheless, the method demonstrates good reproducibility (intermediate precision RSD ≤ 28.4%) across different operators and virus batches, which indicates that it can provide a reliable and repeatable quantitative assessment of AAV-hCYP4V2 biological activity under strict control of key experimental variables.

Compared with the fluorescent substrate-based CYP450 activity assay reported in the literature [17], this method does not require derivatization and enables specific mass spectrometry-based detection in MRM mode, effectively avoiding interference from endogenous fluorescence. In the negative-ion electrospray ionization (ESI) mode, 12-hydroxylauric acid predominantly formed the deprotonated molecular ion [M–H]^−^ at *m*/*z* 215.3. To maximize detection sensitivity and minimize matrix interference, a pseudo-MRM transition (*m*/*z* 215.3 → 215.3) was selected for quantification, thereby avoiding signal loss from inefficient fragmentation. The internal standard, tolbutamide, generated a stable [M–H]^−^ precursor at *m*/*z* 269.0, which upon collision-induced dissociation yielded the characteristic product ion at *m*/*z* 170.1, corresponding to cleavage of the sulfonylurea group. Consequently, the present method provides the absolute quantitation required for GMP potency release, whereas fluorescence-based approaches are not suitable for this purpose. Furthermore, the introduction of an exogenous coenzyme system helps overcome issues related to poor substrate accessibility, thereby enhancing the reaction efficiency.

This method was successfully applied to determine the enzyme activity of AAV2, AAV8, and AAV2/8 mixed serotype products, indicative of its broad applicability. In future studies, this method may be extended to evaluate the activity of other CYP4V2 mutant types or vector systems. This study primarily focuses on in vitro cell lysate-based systems. Although LC-MS/MS has high sensitivity, its automation in clinical trials remains limited, partly due to the complexity of sample pretreatment [18]. Future work may explore the application of this method to in vivo tissue samples or clinical blood samples. In addition, the automation and adaptation of the method for high-throughput analysis represent important directions for future improvement.

## 4. Materials and Methods

### 4.1. Main Instruments

LC-MS/MS analysis was performed using a Shimadzu LMS-8050 triple quadrupole mass spectrometer equipped with a Nexera X2 ultra-high-performance liquid chromatograph (Kyoto, Japan). Cell culture was conducted in a BIOBASE QP-160 carbon dioxide incubator (Jinan, China).

### 4.2. Key Reagents

Lauric acid and its hydroxylated metabolite (12-hydroxydodecanoic acid) were purchased from Macklin (Shanghai, China). Tolbutamide (used as the internal standard for liquid chromatography) was purchased from Solarbio (Beijing, China). Recombinant human cytochrome P450 oxidoreductase (POR) was purchased from R&D Systems (Minneapolis, MN, USA). Human cytochrome b5 and hydroxyurea were purchased from Sigma-Aldrich (St. Louis, MO, USA). Reduced coenzyme II (NADPH) tetrasodium salt was purchased from Sangon Biotech (Shanghai) Co., Ltd (Shanghai, China). DMEM basal medium for cell culture, fetal bovine serum, PBS, trypsin-EDTA, and penicillin–streptomycin were purchased from Gibco (Waltham, MA, USA). The BCA protein assay kit was purchased from Yeasen Biotechnology (Shanghai) Co., Ltd (Shanghai, China). LC-MS grade acetonitrile and methanol were purchased from Fisher Scientific (Waltham, MA, USA). Chromatographically pure ethyl acetate and acetic acid were purchased from DIKMA (Beijing, China).

### 4.3. Virus

Virus samples of rAAV2-hCYP4V2, rAAV8-hCYP4V2, and rAAV2/8-hCYP4V2 were obtained from retained samples of the National Institutes for Food and Drug Control (NIFDC).

### 4.4. Methods

Lauric acid (dodecanoic acid), a representative medium- to long-chain fatty acid, was subjected to ω-hydroxylation, a typical catalytic reaction of the CYP4V2 enzyme [16,19]. This reaction relies on the electron transfer mediated by cytochrome P450 reductase (POR) [20,21,22,23] and cytochrome b5 [24,25,26], as well as the reducing equivalent donor β-nicotinamide adenine dinucleotide phosphate (NADPH) [27]. Therefore, recombinant human POR and human cytochrome b5 were exogenously added to the cell lysate to ensure a maximum reaction rate and accurately reflect CYP4V2 functional activity. The standard procedure is explained below.

#### 4.4.1. Cell Culture, Transduction, and Lysate Preparation

HeLa AAVR cells were pretreated with 0.5 mM hydroxyurea for 24 h, seeded at a density of 3 × 10^6^ cells/dish, and then transduced with rAAV-hCYP4V2 at MOI = 3 × 10^5^ for 120 h. Cells were collected and resuspended in 1 mL of potassium phosphate buffer per 10 cm cell culture dish, followed by ultrasonication. The lysate was then diluted to a final volume of 2 mL.

#### 4.4.2. Enzymatic Reaction System

In a 250 μL reaction system, 200 μL aliquots of cell lysate, P450 reductase (final concentration 40 nM), cytochrome b5 (final concentration 20 nM), and lauric acid substrate (final concentration 250 μM) were added. After incubation at 37 °C for 2 min, NADPH (final concentration 1 mM) was added to initiate the reaction. Reaction times were precisely controlled at 0, 5, 15, 25, and 35 min. Then, 1 M H_2_SO_4_ was added to terminate the reaction, and the samples were stored at −80 °C.

#### 4.4.3. Sample Preparation and LC-MS/MS Conditions

During sample pretreatment, 200 μL of the terminated reaction solution was added with the internal standard. Standard curve and quality control samples were prepared in parallel using blank cell lysate. All samples were subjected to liquid–liquid extraction with ethyl acetate, nitrogen evaporation at 40 °C, and reconstitution in 30% methanol–water before analysis.

The chromatographic separation of 12-hydroxylauric acid and the internal standard (tolbutamide) was performed using an ACE Excel 3 Super C18 column (100 mm × 2.1 mm, 3 μm) maintained at 40 °C. The mobile phase consisted of (A) 0.1% acetic acid in water and (B) acetonitrile, delivered at a flow rate of 0.3 mL/min. The gradient elution program is detailed in Table 2. The total run time was 11.0 min, including column re-equilibration. The injection volume was 5 μL.

Mass spectrometry parameters were as follows: ion source, electrospray; scanning mode, negative ion; detection mode, MRM; spray voltage, −3.0 kV; nebulizing gas flow rate, 3.0 L/min; drying gas flow rate, 10.0 L/min; heating gas flow rate, 10.0 L/min; desolvation line temperature, 250 °C; heating block temperature, 400 °C; interface temperature, 300 °C; ion source interface, Turbo IonSpray (ESI); ionization mode, negative ion; and data acquisition time, 11 min. MRM transitions were optimized for precursor/product ion pairs, and collision energy and other compound-specific parameters were adjusted accordingly. The optimized MRM transitions and conditions are shown in Table 3.

#### 4.4.4. Calibration Curve and Quantification

The calibration curve was constructed using a blank matrix spiked with 12-hydroxylauric acid at concentrations of 0.5, 1, 2, 5, 10, 20, 50, and 100 ng/mL. No background subtraction was applied to the calibration standards. The analyte-to-IS peak area ratios were plotted against nominal concentrations, and a weighted (1/x^2^) least-squares linear regression was performed using LabSolutions software (https://www.shimadzu.com/an/products/software-informatics/index.html, accessed on 20 April 2026). The lowest calibration standard (0.5 ng/mL) was accepted as the lower limit of quantification (LLOQ), with accuracy and precision meeting the acceptance criteria (within ±20% of nominal concentration and RSD ≤ 20%). Values below the LLOQ are reported as “<LLOQ”.

#### 4.4.5. Enzyme Activity Calculation

For study samples, the LC-MS/MS-measured concentrations represent the total 12-hydroxylauric acid present at each time point. To calculate the net product generated by the enzymatic reaction, the concentration measured at time 0 min (reflecting the endogenous baseline prior to NADPH addition) was subtracted from the concentrations measured at all subsequent time points. This subtraction was performed after individual sample concentrations were interpolated from the calibration curve. Enzyme activity was calculated from the slope of the background-corrected concentration–time curve (0–35 min) and expressed as pg/mL/min.

Because the objective of this potency assay is to capture the cumulative efficiency of viral transduction and transgene expression, enzyme activity is reported per unit volume of lysate (pg/mL/min) derived from standardized cell seeding (3 × 10^6^ cells/dish) and lysis (2 mL final volume) procedures. Normalization to total protein content was deliberately avoided, as it would obscure batch-to-batch differences in AAV transduction efficiency—differences that are critical for quality control and product release decisions.

#### 4.4.6. Statistical Analysis

All validation experiments were independently conducted at least three times (*n* ≥ 3). Data are expressed as mean ± standard deviation (SD). Precision was evaluated by the relative standard deviation (RSD), calculated as RSD (%) = (SD/mean) × 100%. For time-course experiments, the significance of the linear relationship between reaction time and product concentration was assessed by linear regression analysis (F-test), with *p* < 0.05 considered statistically significant. Routine statistical calculations were performed using Microsoft Excel 2013. Statistical analyses and graphical representations were generated using GraphPad Prism version 10.1.2.

### 4.5. Methodology Validation

#### 4.5.1. Specificity

Three groups were established: a blank control group without rAAV-hCYP4V2 transduction, a substrate control group containing the enzyme reaction substrate following rAAV-hCYP4V2 transduction, and a positive control group. Each group was subjected to three independent enzyme activity reaction runs.

#### 4.5.2. Accuracy

The accuracy of LC-MS/MS detection was evaluated. Following rAAV-hCYP4V2 transduction, the enzymatic activity assay was performed. After the enzymatic activity reaction was terminated, 100 μL of the samples was collected at three time points and mixed with equal volumes of low-, medium-, and high-level quality control samples to determine the concentrations of 12-hydroxylauric acid and compare it with the theoretical concentration.

#### 4.5.3. Precision

Repeatability was defined as six replicate measurements performed under the same operator within a fixed period according to the standard operating procedure, with RSD values calculated from the measured six enzyme activities.

Intermediate precision was assessed by measuring enzyme activity from three different virus batches analyzed by two operators at different times, with RSD values calculated accordingly.

#### 4.5.4. Linearity and Range

According to the standard operating procedure, enzymatic reaction time points were used as the x-axis, and the 12-hydroxy-octadecanoic acid concentration measured by LC-MS/MS was used as the y-axis. Linear regression was performed using three replicates to evaluate the assay linearity. The analytical range corresponded to the LC-MS/MS calibration curve (0.5–100 ng/mL), covering the detection requirements of this method.

#### 4.5.5. Durability

The effects of different cell passages and different rAAV-hCYP4V2 batches on enzyme activity measurement were evaluated. Meanwhile, the stability of test samples stored at −80 °C for 4 weeks after the completion of the enzymatic reaction was evaluated.

### 4.6. Application of Methods

To verify the applicability of the established method and its ability to distinguish differences in activities of different products, the method was applied in the quantitative analysis of CYP4V2 enzyme activity in three rAAV-hCYP4V2 gene therapy products, including an AAV8 serotype vector (rAAV8-hCYP4V2), an AAV2 serotype vector (rAAV2-hCYP4V2), and a hybrid serotype research product (rAAV2/8-hCYP4V2). All products were tested according to the standard operating procedures described in Section 2.4, with three independent replicates (*n* = 3) for each product. By comparing mean enzyme activity, coefficient of variation (RSD), and statistical differences among products, the applicability of the method for product quality control and comparative analysis was evaluated.

## Figures and Tables

**Table 1:** The application results of the established method.

Product	Average Enzyme Activity (pg·mL^−1^·min^−1^)	Standard Deviation	RSD (%)
rAAV8-hCYP4V2	328.4	78.51	23.91
rAAV2-hCYP4V2	150.7	24.26	16.10
rAAV2/8-hCYP4V2	159.7	34.39	21.53

Note: All data are based on 3 independent replicates.

**Table 2:** Liquid chromatography gradient elution program.

Time (min)	Mobile Phase B (%)
0.00	40
6.00	76
6.10	99.9
8.00	99.9
8.10	40
11.00	40 (Stop)

Note: Mobile phase A was 0.1% acetic acid in water; mobile phase B was acetonitrile. The flow rate was maintained at 0.3 mL/min throughout the run.

**Table 3:** Ion-pair parameters of 12-hydroxylauric acid and tolbutamide.

No.	Compound Name	Precursor Ion	Product Ion	Q1 Pre Bias (V)	CE	Q3 Pre-Bias (V)
1	12-Hydroxylauric acid	215.3	215.3 *	22	9	16
			169.3	10	18	18
			197.2	15	17	20
2	Tolbutamide	269	170.1	28	18	18

Note: The transition *m*/*z* 215.3 → 215.3 represents a pseudo-MRM transition used for the quantification of 12-hydroxylauric acid, as it provided a superior signal-to-noise ratio and stability compared with fragmentation-based transitions under the optimized source conditions. The asterisk (*) denotes the quantifier ion transition used for peak integration and concentration calculation.

## Data Availability

The data presented in this study are available upon reasonable request from the first author.

## Supplementary Materials

The following supporting information can be downloaded at: https://www.mdpi.com/article/10.3390/molecules31091417/s1, Table S1: 12-Hydroxylauric acid concentration in enzymatic reactions after rAAV-hCYP4V2 transduction in HeLa-AAVR cells; Table S2: Product concentration and corresponding enzyme activity under different combinations of cell seeding number and MOI; Table S3: Concentrations of 12-hydroxylauric acid detected at different enzymatic reaction times; Table S4: Specificity evaluation results; Table S5: Accuracy evaluation results; Table S6: Repeatability evaluation results; Table S7: Intermediate precision evaluation results; Table S8: Linearity evaluation results; Table S9: Stability evaluation results.

## Abbreviations

The following abbreviations are used in this manuscript:

AAV	Adeno-associated virus
BCA	Bicinchoninic acid assay
BCD	Bietti crystalline dystrophy
CE	Collision energy
CYP4V2	Cytochrome P450 family 4 subfamily V member 2
DMEM	Dulbecco’s modified Eagle medium
EDTA	Ethylenediaminetetraacetic acid
ESI	Electrospray ionization
HPLC	High-performance liquid chromatography
HU	Hydroxyurea
ICH	International Council for Harmonisation
LC-MS/MS	Liquid chromatography–tandem mass spectrometry
LLOQ	Lower limit of quantification
MOI	Multiplicity of infection
MRM	Multiple reaction monitoring
NADPH	Nicotinamide adenine dinucleotide phosphate (reduced form)
NIFDC	National Institutes for Food and Drug Control
PBS	Phosphate-buffered saline
POR	Cytochrome P450 oxidoreductase
Q1 Pre Bias	Quadrupole 1 pre-bias
Q3 Pre Bias	Quadrupole 3 pre-bias
*R*^2^	Coefficient of determination
rAAV-hCYP4V2	Recombinant adeno-associated virus carrying the human *CYP4V2* gene
RPE	Retinal pigment epithelium
RSD	Relative standard deviation
SD	Standard deviation
