# Supplementary material for: Development and Validation of a Quantitative LC-MS/MS Method for Measuring CYP4V2 Enzyme Activity via 12-Hydroxylauric Acid in rAAV-hCYP4V2 Gene Therapy Products
Source: Molecules. 2026 Apr 24;31(9):1417. doi: 10.3390/molecules31091417 (PMC13165093; doi:10.3390/molecules31091417)
Supplement: Supplementary file 1 [file molecules-31-01417-s001.zip › molecules-4238686-supplementary.pdf]

Development and validation of a quantitative LC-MS/MS method for measuring CYP4V2 enzyme activity via 12-hydroxylauric acid in rAAV-hCYP4V2 gene therapy products

Ge Ren[1,2,3†], Xi Qin[1,2†], Yiran Li[1,2†], Wenhong Fan[1,2], Wenjing Luo[4], Yanrong Cao[4], Yang Wang[3*], Yong Zhou[1,2*], Chenggang Liang[1,2*]

Table S1. 12-Hydroxylauric acid concentration in enzymatic reactions after rAAV-hCYP4V2 transduction in HeLa-AAVR cells.

HU=0			HU=0.5 mM		
Time (min)	Product concentration (ng/mL)	Product concentration after background subtraction (ng/mL)	Time (min)	Product concentration (ng/mL)	Product concentration after background subtraction (ng/mL)
0	4.17	0	0	3.33	0
15	6	1.83	15	6.97	3.64
30	6.07	1.9	30	7.78	4.45
45	6.71	2.54	45	9.42	6.09
60	5.51	1.34	60	9.44	6.11
75	5.21	1.04	75	4.17	0.84
90	5.38	1.21	90	5.81	2.48
105	3.22	LLOQ	105	9.11	5.78
120	4.79	0.62	120	10.11	6.78

Note: Values reported as “<LLOQ” indicate concentrations below the lower limit of quantification (0.5 ng/mL) after background subtraction. Negative values arising from subtraction of the 0-min time point were also replaced with “<LLOQ”.

Table S2. Product concentration and corresponding enzyme activity under different combinations of cell seeding number and MOI.

No.	HeLa-AAVR cell seeding number	Virus MOI	Product concentration at 0 min after enzymatic reaction (ng/mL)	Product concentration at 45 min after enzymatic reaction (ng/mL)	Background subtraction (ng/mL)	Corresponding enzyme activity (pg/mL/min)
1	1 x 10^6	3 x 10^4	4.01	4.31	0.3	6.67
2	1 x 10^6	1 x 10^5	3.96	LLOQ	LLOQ	0
3	1 x 10^6	3 x 10^5	4.49	5.9	1.41	31.33
4	3 x 10^6	3 x 10^4	4.72	7	2.28	50.67
5	3 x 10^6	1 x 10^5	5.33	14.01	8.68	192.89
6	3 x 10^6	3 x 10^5	6.01	18.12	12.11	269.11
7	5 x 10^6	3 x 10^4	7.4	7.23	LLOQ	0
8	5 x 10^6	1 x 10^5	5.63	13.72	8.09	179.78
9	5 x 10^6	3 x 10^5	6.02	14.71	8.69	193.11

Note: Concentrations reported as '<LLOQ' are below the lower limit of quantification (0.5 ng/mL). Enzyme activity calculated from these values is reported as 0 pg/mL/min to indicate no detectable activity under these conditions.

Table S3. Concentrations of 12-hydroxylauric acid detected at different enzymatic reaction times.

reaction time (min)	Product concentration after background subtraction (ng/mL)
0	0
5	0.32
15	3.99
25	4.33
35	5.21
45	3.28
55	3.64
65	4.75
90	4.65
120	5.33

Table S4. Specificity evaluation results.

Experimental group	Replicate	Time (min)	Concentration of 12-hydroxylauric acid after background subtraction (ng/mL)	Enzyme activity (pg/mL/min)
Blank control group	Dish 1	0	0.00	NA
		5	LLOQ	
		15	LLOQ	
		25	LLOQ	
		35	LLOQ	
	Dish 2	0	0.00	NA
		5	0.23	
		15	0.06	
		25	0.00	
		35	0.12	
	Dish 3	0	0.00	NA
		5	0.00	
		15	0.00	
		25	0.75	
		35	0.56	
Substrate (acetic acid) group	Dish 4	0	0.00	NA
		5	0.00	
		15	0.00	
		25	0.00	
		35	0.59	
	Dish 5	0	0.00	NA
		5	0.00	
		15	0.00	
		25	0.00	
		35	0.00	
	Dish 6	0	0.00	NA
		5	0.00	
		15	0.00	
		25	0.52	
		35	0.52	
Positive control group	Dish 7	0	0.00	147.6
		5	0.85	
		15	2.12	
		25	4.14	
		35	4.87	
	Dish 8	0	0.00	160.7
		5	0.96	
		15	2.21	
		25	4.41	
		35	5.41	
	Dish 9	0	0.00	142.4
		5	0.75	
		15	2.45	
		25	2.70	
		35	5.46	

Table S5. Accuracy evaluation results.

Replicate	Time (min)	Concentration of 12-hydroxylauric acid (ng/mL)	Spiked concentration (ng/mL)	Theoretical concentration (ng/mL)	Measured concentration (ng/mL)	Accuracy %
Dish 1	0	0.56	75.00	37.78	33.00	87.3
	15	2.68	30.00	16.34	17.34	106.1
	35	5.43	1.50	3.465	3.57	103.0
Dish 2	0	0.62	75.00	37.81	39.03	103.2
	15	2.83	30.00	16.415	16.98	103.4
	35	6.03	1.50	3.765	3.63	96.4
Dish 3	0	0.58	75.00	37.79	36.57	96.8
	15	3.03	30.00	16.515	17.38	105.2
	35	6.04	1.50	3.77	3.92	104.0

Table S6. Repeatability evaluation results.

Replicate	Time (min)	Concentration of 12-hydroxylauric acid after background subtraction (ng/mL)	Enzyme activity (pg/mL/min)
Dish 1	0	0.00	147.6
	5	0.85	
	15	2.12	
	25	4.14	
	35	4.87	
Dish 2	0	0.00	160.7
	5	0.96	
	15	2.21	
	25	4.41	
	35	5.41	
Dish 3	0	0.00	142.4
	5	0.75	
	15	2.45	
	25	2.70	
	35	5.46	
Dish 4	0	0.00	67.2
	5	0.00	
	15	0.95	
	25	1.66	
	35	2.44	
Dish 5	0	0.00	52.0
	5	0.52	
	15	0.85	
	25	1.19	
	35	1.83	
Dish 6	0	0.00	60.0
	5	0.51	
	15	0.82	
	25	1.39	
	35	2.18	
Dish 7 Operator B	0	0.00	182.5
	5	0.80	
	15	2.63	
	25	4.55	
	35	6.46	
Dish 8 Operator B	0	0.00	174.5
	5	0.79	
	15	2.61	
	25	4.48	
	35	6.04	
Dish 9 Operator B	0	0.00	184.6
	5	0.99	
	15	2.68	
	25	4.63	
	35	6.48	

Note: The three dishes were grouped, and the cell passages used were P16, P20, and P19, respectively. All virus samples were from batch V1.

Table S7. Intermediate precision evaluation results.

Replicate group	Time (min)	Concentration of 12-hydroxylauric acid after background subtraction (ng/mL)	Enzyme activity (pg/mL/min)
Dish 10	0	0.00	180.7
	5	1.54	
	15	2.84	
	25	4.36	
	35	6.29	
Dish 11	0	0.00	110.5
	5	0.80	
	15	1.88	
	25	2.59	
	35	3.86	
Dish 12	0	0.00	142.7
	5	0.92	
	15	2.08	
	25	3.39	
	35	5.12	
Dish 13	0	0.00	83.7
	5	0.91	
	15	1.51	
	25	1.97	
	35	2.84	
Dish 14	0	0.00	106.8
	5	0.62	
	15	1.53	
	25	3.03	
	35	3.50	
Dish 15	0	0.00	106.1
	5	0.88	
	15	1.68	
	25	2.49	
	35	3.74	

Note: The cell passage used in dishes 10 to 12 was P16, and the virus batch was V2. The cell passage used in dishes 13 to 15 was P15, and the virus batch was V3.

Table S8. Linearity evaluation results.

Replicate	R^2
Dish 1	>0.999
Dish 2	>0.999
Dish 3	>0.999
Dish 4	0.987
Dish 5	0.995
Dish 6	0.976
Dish 7	0.991
Dish 8	0.985
Dish 9	0.991
Dish 10	0.994
Dish 11	0.995
Dish 12	0.998
Dish 13	0.978
Dish 14	0.992
Dish 15	0.993

Table S9. Stability evaluation results.

Replicate	Time (min)	Concentration of 12-hydroxylauric acid (ng/mL)	Enzyme activity (pg/mL/min)	Re-measured values after four weeks	Enzyme activity (pg/mL/min)	Enzyme activity recovery rate (%)
Dish 4	0	LLOQ	67.2	LLOQ	68.4	101.8
	5	LLOQ		0.64		
	15	0.95		1.12		
	25	1.66		1.6		
	35	2.44		2.39		
Dish 5	0	LLOQ	52.0	LLOQ	57.9	111.3
	5	0.52		0.56		
	15	0.85		0.91		
	25	1.19		1.24		
	35	1.83		2.12		
Dish 6	0	LLOQ	60.0	LLOQ	55.3	92.2
	5	0.51		0.52		
	15	0.82		0.96		
	25	1.39		1.28		
	35	2.18		1.92		

Note: LLOQ indicated values below the quantification limit.
